# Evaluating the effect of insulin sensitizers metformin and pioglitazone alone and in combination on women with polycystic ovary syndrome: An RCT

**Published:** 2016-12

**Authors:** Seyed Mojtaba Sohrevardi, Fahime Nosouhi, Saeed Hossein Khalilzade, Parichehr Kafaie, Mojgan Karimi-Zarchi, Iman Halvaei, Mehdi Mohsenzadeh

**Affiliations:** 1 *Faculty of Pharmacy, Shahid Sadoughi University of Medical Sciences and Health Services, Yazd, Iran.*; 2 *Division of Endocrinology, Department of Internal Medicine, Shahid Sadoughi University of Medical Sciences and Health Services, Yazd, Iran.*; 3 *Department of Dermatology, Shahid Sadoughi University of Medical Sciences and Health Services, Yazd, Iran.*; 4 *Departments of Obstetrics and Gynecology, Shahid Sadoughi University of Medical Sciences and Health Services, Yazd, Iran.*; 5 *Recurrent Abortion Research Center, Research and Clinical Center for Infertility, Shahid Sadoughi University of Medical Sciences, Yazd, Iran* *.*; 6 *Department of Anatomical Sciences, Faculty of Medical Sciences, Tarbiat Modares University, Tehran, Iran.*; 7 *Research and Clinical Center for Infertility, Shahid Sadoughi University of Medical Sciences, Yazd, Iran* *.*

**Keywords:** *Polycystic ovary syndrome*, *Metformin*, *Pioglitazone*, *Insulin resistance*

## Abstract

**Background:**

Insulin resistance and hyperinsulinemia may play a role in pathogenesis of PCOS. One of the common therapeutic methods is using insulin-sensitizing drugs such as metformin and thiazolidinediones.

**Objective:**

The purpose was to determine the effect of metformin and pioglitazone on clinical, hormonal and metabolic parameters in women with PCOS.

**Materials and Methods:**

Eighty four women randomly received one of the following for 3 months: metformin (n=28) (500 mg three times a day), pioglitazone (30 mg daily) (n=28) and combination of both metformin and pioglitazone (n=28) (30 mg/day pioglitazone plus 500 mg metformin three times a day). Hormonal profile, fasting serum insulin, body weight, body mass index, menstrual status and waist to hip ratio were evaluated before and after treatment.

**Results:**

Metformin and pioglitazone and combination therapy induced favorable changes in fasting serum insulin, HOMA-IR index, QUICKI, fasting glucose to insulin ratio in women with PCOS. Body weight, BMI, and waist to hip ratio increased significantly after treatment with pioglitazone but the data were similar after administration of metformin or combination therapy. Total testosterone level decreased significantly only after treatment with metformin. After 3 months in patients who received pioglitazone or combination therapy, menstrual cycles became regular in 71.4% and 73.9% respectively. While menstrual improvement happened only in 36.4% of the patients treated with metformin.

**Conclusion:**

These findings suggest that insulin-sensitizing drugs induce beneficial effect in insulin resistance and menstrual cyclicity but only metformin ameliorated hyperandrogenemia in women with PCOS. Treatment with combination of metformin and pioglitazone did not show more benefit than monotherapy with each drug alone.

## Introduction

Polycystic ovary syndrome (PCOS) is one of the most common endocrinological disorders diagnosed by detection of at least two of the following criteria; polycystic ovaries on ultrasound, menstrual irregularities, hyperandrogenism based on biochemical and/or clinical manifestation (1). Insulin resistance and hyperinsulinemia may play an important role in pathogenesis of PCOS and overweight in PCOS women (2, 3). It has been reported that insulin resistance and consequent hyperinsulinemia play a prominent role in hyperandrogenemia through direct way, induction of androgen production by theca cells, and indirect ways like elevation of luteinizing hormone (LH) secretion, decreasing insulin-like growth factor (IGF) binding protein, and reducing hepatic synthesis of sex hormone binding globulin (SHBG) and consequently free androgen level augmentation occurs (4-6). Hyperinsulinemia and insulin resistance in PCOS patients appears to simulate or aggravate hyperandrogenism because of gonadotropin secretory disturbance (2).

It has been postulated that abnormalities in insulin action (both insulin resistance and hyperinsulinemia), contribute to important comorbidities including: glucose intolerance, type 2 diabetes mellitus (DM2), dyslipidemia, increased plasma triglycerides and decreased plasma high-density lipoprotein cholesterol (HDL-C), hypertension, and atherosclerosis (7, 8). Likewise, insulin resistance has adverse effect on fertility and pregnancy rate (9, 10). PCOS is also associated with increased risk of endometrial cancer (11).

Since there is a relationship between PCOS and insulin resistance, administration of drugs to ameliorate insulin sensitivity and ovarian activity is suggested to treat this syndrome (12). Various clinical studies have focused on two different types of insulin-sensitizing drugs for treatment of women with PCOS. Metformin and thiazolidinediones (TZD) (troglitazone, rosiglitazone, pioglitazone) are standard medications for treatment of DM2 via decreasing insulin resistance, improving insulin sensitivity in peripheral tissues, reducing free androgen levels and facilitating normal regular menses and pregnancy (13-16).

Metformin inhibits hepatic glucose production and lipogenesis, and also increases peripheral glucose uptake and reduces fatty acid oxidation as well. Pioglitazone, as a peroxisome proliferator-activated receptor γ agonist, decreases insulin resistance in liver and peripheral tissue and has anti-arteriosclerotic and anti-inflammatory effects. Moreover, probably pioglitazone decreases cardiovascular risk factors (17-19). Many investigations have shown that metformin reduces insulin resistance and endocrine parameters in women with PCOS and is considered the most used drug to treat PCOS patients (20-25).

Although these two groups of insulin sensitizers have been shown to be effective, but they do not entirely restitute normal reproductive phenotype. Many women with PCOS do not response optimally to insulin sensitization even at the highest doses. Metformin and TZDs are also used as a combination therapy due to their "add-on effect" on insulin resistance; therefore it seems that combination therapy is required to treat all features of PCOS.

So far, few investigations have performed on treatment of PCOS with pioglitazone and also few studies have compared these insulin sensitizers alone or in combination in patients with PCOS (26, 27). Until now, it has not been proved which group of insulin sensitizer drugs is more efficient and suitable for initiation of PCOS treatment. Another question is whether combination therapy with two different types of insulin-sensitizer drugs is more potent than a single agent therapy (27). Also the effects of pioglitazone and metformin alone and in combination in three groups have not been reported in Middle Eastern women with PCOS. 

The main goal of the current study was to compare the clinical, hormonal and metabolic change after treatment with metformin and pioglitazone. The specific goals were to distinguish whether the combination of the pioglitazone and metformin might be more effective in improvement menstrual regularity, hyperandrogenemia and prevention of long-term health hazard than using each drug alone.

## Materials and methods

**Subjects**

Eighty four women with PCOS, aged 18-40 years old, with irregular menses and infertility and/or clinical sign of hyperandrogenism (hirsutism and acne) were recruited from Department of Gynecology, Shahid Sadoughi Hospital Yazd, Iran. between April 2014 to May 2015. Written informed consent was obtained from all participants. The research protocol was approved by the Ethics Committee of the Shahid Sadoughi University of Medical Sciences.

All patients had spontaneous inception of puberty and normal sexual growth and did not take oral contraceptive pills or anti-androgens. Also none of patients had received any drugs affecting hormonal profile and lipoprotein or glucose metabolism, or appetite at least three month before the onset of study.

The diagnosis of PCOS was based on at least two of the three following abnormalities according to the 2003 Rotterdam Consensus Conference: 1) Oligomenorrhoea or amenorrhea; Oligomenorrhea was defined as menstrual cycle with an interval of ≥35 days. Secondary amenorrhea was defined as the absence of mense for at least six months. Irregular menstrual cycles was defined as cycle with changes of the intermenstrual interval of more than ±4 days and a period of ≤35 days (28). Regular menstrual was defined as cycles with the minimum and maximum length change of less than four days and cycle length ranging from 21-35 days, 2) Manifestation of hyperandrogenemia such as hirsutism (Ferriman-Gallwey Score ≥8), acne and/or serum total testosterone greater than 70 ng/dL, 3) Polycystic ovaries by ultrasonography; confirmed by presence of 12 or more subcapsular follicles measuring 2-9 mm in diameter at least in one ovary or increased ovarian volume (>10 cm^3^) (29-31).

Patients with thyroid dysfunction, hyperprolactinemia, Cushing’s syndrome, any androgen-secreting tumors, DM (fasting plasma glucose >7 mmol/L), nonclassical 21-hydroxylase deficiency, autoimmune disease, central nervous system disease, significant hypertension, past hysterectomy, abnormal liver or kidney functions or active liver disease (ALT >2.5 the upper limit of normal range), and known heart disease, pregnancy and lactation were excluded from the study. Lost to follow-up, diagnosis of pregnancy and elevated levels of serum transaminases were exclusion criteria during the study. 

**Study design**

Eighty four PCOS women were randomly assigned to one of the following groups during three months: group 1 consisted 28 patient received 500 mg metformin (t.i.d) with meals, group 2 consisted 28 subject received 30 mg pioglitazone (daily) and group 3 consisted of 28 subjects, received combination of pioglitazone 30 mg (daily) and metformin 500 mg (t.i.d). To reduce gastrointestinal side effect of metformin in the first wk of therapy, the dose of metformin was increased stepwise, from 500 mg with the evening meal for two days to 500 mg twice a day for two days and thereafter 500mg three times per day. Before initiation of the study, all patients were allocated to three different groups in sequences provided by computer program generating random number. All subjects received a sealed envelope including the number 1 or 2 or 3 which was corresponding to metformin, pioglitazone and combination therapy, respectively. In our study because commercially accessible pills were applied, there was no blinding after randomization, therefore, investigator and subjects could be aware of the actual treatment.

Before the study, cortisol levels for all patients tested to rule out Cushing's syndrome and its impact on irregular menstruation, at 8 am To evaluate the drags effect on BMI, height and weight, waist-to-hip ratio for all patients was measured before and after the study. All subjects were on unrestricted diet and were advised not to change their physical exercise patterns during the study period. During the study, monthly follow-up visits were performed for all patients to evaluate menstrual status, side effects of the drugs and patients compliance. Compliance was monitored by the self-reporting of the patients. Menstrual period length and cyclicity were evaluated by a self-filled menstrual calendar.

All potentially fertile patients were advised to use barrier method of contraception during the study if pregnancy was not desired and they were cautiously instructed to stop using the drug by confirmation of pregnancy. At the beginning of the study and after three months of treatment, in the early follicular phase (day 3-5) of spontaneous bleeding or progestin-induced withdrawal bleeding, venous blood samples were collected after a 10-12h overnight fast to measure luteinizing hormone (LH), follicle-stimulating hormone (FSH), fast glucose, fast insulin, dehydroepiandrosterone sulfate (DHEA-S), total testosterone (T), total cholesterol (TC), triglycerides (TG), high-density lipoprotein (HDL) cholesterol, low-density lipoprotein (LDL) cholesterol, alanine aminotransferase (ALT), aspartate aminotransferase (AST) and complete blood count. Thyroid-stimulating hormone (TSH), prolactin (PRL), 17-hydroxyprogesterone (17OHPG) and plasma cortisol were evaluated in order to exclude other disorder with similar feature to PCOS.

Also height, weight, body mass index (BMI), waist to hip ratio, Ferriman-Gallwey score, acne score and menstrual history were recorded for all participants. Acne was assessed in four scores; 0= no acne; 1= slight acne on the face only; 2= moderate acne on the face only; 3= severe acne on the face and back or chest (32). All examinations were performed by the same practitioner. 

Women were instructed to record a menstrual diary by a self-filled menstrual calendar during the study period to evaluate menstrual period length and cyclicity. The waist and hip circumferences were measured with a soft tape measure pursuant to the World Health Organization criteria. To measure plasma cortisol, blood sample was obtained at 8 A.M. and it was in the normal range for all subjects. In all patients, congenital adrenal hyperplasia was excluded by measurement of morning follicular phase 17-hydroxyprogesterone greater than 2 ng/ml.

At the base line of study (0 week) and after 3 months of treatment, women of all groups underwent ultrasonography in order to measure the number and size of follicles and ovarian size; Volume measurements were made by using the formula for the volume of an ellipsoid: 0.523 × length ×width × thickness (33). In this investigation, insulin resistance was evaluated by different methods consisted of the homeostasis model assessment (HOMA), the quantitative insulin sensitivity check index (QUICKI) and fasting glucose to fasting insulin ratio (34, 35). HOMA was calculated in the following way:

fasting serum insulin μUml×fasting plasma glucose (mmol1)

The QUICKI was calculated using the formula:

$\frac{1}{\left[\log(\mathrm{fastin}\mathrm{g serum insulin}\left(\frac{\mathrm{\mu U}}{\mathrm{ml}}\right)+\log\mathrm{fasting plasma glucose}(\frac{\mathrm{mg}}{\mathrm{dl}})\right]}$

**Laboratory assay**

Nonheparinized venous blood samples were centrifuged, and serum was stored at -20^o^C until analysis. Fasting serum insulin was determined using chemiluminescence immunoassay (Insulin AccuLite CLIA Kit, USA). Serum fasting glucose was determined using a glucose oxidase method and using BT-3000 PLUS analyzer, Italy. The intra- and inter-assay coefficients of variation were ≤1.74% and ≤1.19%, respectively.

Testosterone and DHEAS was measured using chemiluminescence immunoassay by *DiaSorin LIAISON*® Analyzer. For testosterone the intra- and inter-assay coefficients of variation were ≤5%% and ≤10%, respectively. LH, FSH and PRL were determined by chemiluminescence immunoassay (AccuLite CLIA Kit, Monobindlnc, Lake Forest, CA92630, USA). For LH the intra- and inter-assay coefficients of variation were ≤7.9% and ≤9.5%, respectively. For PRL the intra- and inter-assay coefficients of variation were ≤4.3%and≤9.8%, respectively.

Cortisol was measured by *radioimmunoassay (RIA kit) and *TSH was measured using immunoradiometric assay (RIAKEY® TSH IRMA Tube) with DREAM GAMMA-10 analyzer and the intra- and inter-assay coefficients of variation were ≤5.1% and ≤8.21%, respectively. 17OHP was measured by enzyme immunoassay (AccuBind ELISA kit, Monobindlnc, Lake Forest, CA92630, USA). The intra- and inter-assay coefficients of variation were ≤8.5 and ≤8%, respectively. Spectrophotometry was used to determination of HDL, TG, total cholesterol, ALT and AST by using BT-3000 PLUS analyzer, Italy. The intra- and inter-assay coefficients of variation of lipids profile were less than 5%.

**Statistical** **analysis**

All outcome measures were represented as mean±SD. In this study, Wilk-Shapiro test was performed to assess the normality of the distribution of variables. Student’s two-tailed paired *t*-test or the Wilcoxon signed rank test were used to compare mean variables at baseline to mean values after treatment within groups. One way ANOVA and post-hoc Bonferroni, were carried out to compare means between three groups. Chi-square and Fisher’s exact test were used to evaluate menstrual pattern. Acne score and hirsutism score data had skewed distribution, so Kruskal-Wallis test was used for comparison of differences between groups. These data were reported as median and quartile range. 

Because there was significant difference at the baseline in fasting glucose between three groups, we used analysis of covariance (ANCOVA) for comparison fasting glucose before and after treatment. A two tailed p*<*0·05 was considered statistically significant. In this study the SPSS statistical software package (version 22.0 for Windows; SPSS, Inc. Chicago, IL) was used. In RCTs, it is necessary to mention how the authors calculated the sample size.

## Results

**Baseline characteristics**

**Figure 1:**
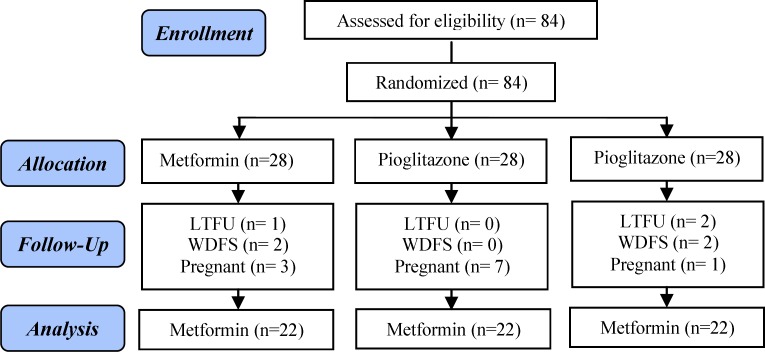
Chart of the study from recruitment to completion after three months of treatment.

In the metformin group, two subjects withdrew from the study because of severe gastrointestinal side effects. One woman was lost to follow up, and three became pregnant. In the pioglitazone group, seven women became pregnant and in the combination group two women were lost to follow up, two patients withdrew from the study because of intolerable gastric discomfort and one became pregnant. The clinical, metabolic, and hormonal measures at the baseline evaluation did not show significant differences between women who became pregnant or were lost to follow-up (Figure 1).

Sixty six women (age 29.04±5.93 years old) with BMI of 27.80±3.89 (kg/m^2^) finished the trial and were included in final statistical analysis. Pioglitazone was well tolerated and no patients discontinued pioglitazone because of side-effects. During the study period, no raise in serum transaminases were detected. There were no significant differences at the baseline in metabolic, clinical and hormonal measures between the three groups (ANOVA test) but baseline fasting glucose was higher in the metformin group than in other groups (p=0.014) and consequently baseline HOMA-IR index was higher in metformin group (p=0.04**)** (Table I).

In metformin group, 11 (50%) and 5 (22.7%) subjects, in pioglitazone group, 8 (40%) and 6 (30%) and in combination group 12 (52.2%) and 8 (34.8%) were overweight and obese, respectively. All patients had polycystic ovaries appearing on ultrasonography. Before treatment, 11 patients were amenorrheic (4 in metformin group, 4 in pioglitazone, 3 in combination group); 29 women were oligomenorrheic (10 in metformin group, 8 in pioglitazone group, 11 in combination group); 14 women had irregular menses and 12 women had regular menstrual cycle.

All women were affected by clinical and/or biochemical hyperandrogenism. An F-G score ≥8 was detected in 56 patients, 86.3% in metformin group, 76.1% in pioglitazone group and 91.3% in combination group. Acne was observed in 72.7% of patients and hyperandrogenemia (defined as total testosterone >70 ng/ml) in 36.3% of subjects. Fasting hyperinsulinemia (>16 U/ml) was detected in 30.3%.

**Post-treatment characteristics**

**Metabolic parameters**

Weight were increased significantly only in pioglitazone group (1.5 kg, p<0.001). Likewise, BMI was significantly higher in the pioglitazone group (p<0.001) after 3-month treatment but BMI and weight remained unchanged in metformin group and combination therapy. Waist to hip ratio was increased significantly (p=0.001) after pioglitazone treatment and remained unchanged after metformin treatment or combination therapy. None of the women had DM at the baseline. 

Treatment with metformin resulted in significant decreases in fasting glucose concentration (p=0.012), fasting insulin (p=0.003), and the HOMA-IR index (p=0.001). QUICKI (p=0.001) and fasting glucose to insulin ratio (p=0.008) was increased significantly after metformin treatment. The treatment with pioglitazone decreased fasting glucose concentration (p=0.018), fasting insulin (p=0.015), and HOMA-IR index (p=0.011). Pioglitazone treatment increased significantly QUICKI (p=0.007) and fasting glucose to insulin ratio (p=0.026).

After treatment with combination of metformin and pioglitazone, fasting insulin (p=0.026) and HOMA-IR index (p=0.013) decreased significantly. QUICKI (p=0.009) and fasting glucose to insulin ratio (p=0.03) were significantly increased. Fasting glucose showed decreasing trend but the difference was not significant. There were no differences for fasting insulin levels, insulin sensitivity indices (HOMA, QUICKI and fasting glucose to insulin ratio) between three groups after treatment (p=0.144, p=0.084, p=0.33 and p=0.82, respectively). Serum concentrations of TG, TC, LDL and HDL cholesterol, were similar between groups at the baseline*,* within the normal ranges and remained unchanged after treatment.

**Hormonal parameters**

At the baseline, the hormonal parameters were similar in all groups. LH level as well as the ratio of LH to FSH were decreased significantly (p=0.002) but there was no significant change in the FSH levels after treatment with metformin. Pioglitazone and combination therapy had similar effects on pituitary hormones, causing significant increases in the FSH level (p=0.041 and p=0.005, respectively) and no significant change in the LH level was observed. The ratio of LH to FSH significantly decreased in both pioglitazone and combination group (p=0.007 and p=0.003, respectively). There was a decrease in serum total testosterone (p=0.018) after treatment with metformin but no significant differences were observed after pioglitazone and combination therapy. After treatment, subject who received metformin or pioglitazone or combination therapy did not indicate significant change in final serum DHEAS.

**Menstrual pattern**

During three months of administration of metformin and pioglitazone, 11 pregnancies were occurred (3 pregnancies on metformin, 7 on pioglitazone and 1 on combination therapy). This suggests that these drugs, particularly pioglitazone, result in rapid induction of regular menstrual cycles and ovulation in a subset of these women. There was a striking amelioration in menstrual cyclicity among the patients who were received pioglitazone or combination therapy.

In patients with menstrual disturbance treated with pioglitazone and combination therapy, menstrual cycles became regular in 71.4% and 73.9% respectively. While improvement happened in 36.4% of the patients treated with metformin. There was a significant variation between metformin group and combination therapy in regularity of menstrual cycles (p=0.031)

After three months of treatment in pioglitazone group, 6 of 8 oligomenorrheic and 1 of 4 amenorrheic women achieved regular cycles, and 3 women with irregular menses attained regular cycles. Three women still had amenorrhea. In combination therapy, 9 of 11 oligomenorrheic and 1 of 3 amenorrheic subjects became eumenorrhiec, and 3 of 5 women with irregular menstrual cycles achieved regular menses. Two women remained in amenorrhea. In metformin group, 1 of 10 women who had oligomenorrhea and 3 of 5 irregular menses achieved regular cycles and 1 of 4 amenorreheic became oligomenorrheic. 11 women who were oligo- or amenorrheic at baseline reported no improvement in menstrual pattern.

**Hyperandrogenism clinical manifestations**

In our study, the administration of metformin, pioglitazone and combination therapy resulted in a significant decrease in the acne score by 38.9% (p=0.002), 68.4% (p<0.001) and 76.1%9 (p=0.001) respectively. No significant decrease was observed in the hirsutism score during the course of the study in three groups.

**Table I:** Baseline characteristics of women with PCOS

**Characteristics**	**Metformin group** **(N=22)**	**Pioglitazone group** **(N=21)**	**Combination group** **(N=23)**	**p-value**
Age	28.72 ± 6.31	27.52 ± 5.01	30.73 ± 6.15	0.19
Weight (kg)	71.3 ± 11.2	70.6 ± 13.2	72.6 ± 9.4	0.82
BMI (kg/m^2^)	27.5 ± 3.6	27.2 ± 4.7	28.5 ± 3.2	0.54
Waist-to-hip ratio	0.8 ± 0.03	0.8 ± 0.05	0.8 ± 0.04	0.17
Fasting glucose (mmol/l)	5.4 ± 0.5	5 ± 0.2	5.1 ± 0.5	0.014
Fasting insulin (µU/ml)	17.1 ± 10.8	12.4 ± 7.1	11.7 ± 7.5	0.08
HOMA-IR	4.2 ± 2.7	2.8 ± 1.6	2.7 ± 1.8	0.04
QUICKI	0.3 ± 0.03	0.3 ± 0.02	0.3 ± 0.03	0.05
Fasting G/I ratio (mg/dL per µIU / mL)	7.8 ± 5.3	9.5 ± 4.7	10.6 ± 6.3	0.25
LDL cholesterol (mg/dl)	131.8 ± 26.7	113.8 ± 54.8	126.9 ± 33.5	0.28
HDL cholesterol (mg/dl)	51.1 ± 12.1	51.1 ± 12.5	52.2 ± 12.2	0.94
Serum cholesterol (mg/dl)	215 ± 36.5	185.6 ± 51.6	196 ± 36.6	0.07
Serum triglyceride (mg/dl)	140.3 ± 58.2	111.6 ± 76	131 ± 59.3	0.34
DHEAS (mg/l)	1.5 ± 0.7	1.4 ± 0.7	1.4 ± 0.7	0.79
Total testosterone (µg/l)	0.7 ± 0.2	0.7 ± 0.1	0.6 ± 0.2	0.58
FSH (IU/L)	5.2 ± 1.7	4.7 ± 1.2	5.6 ± 2.2	0.34
LH(IU/L)	7.9 ± 2.3	7.6 ± 2.9	6.8 ± 2.7	0.37
LH/FSH	1.6 ± 0.6	1.6 ± 0.8	1.4 ± 0.7	0.48

Note*: *Plus-minus values are means ± SD. To convert values for fasting glucose to mmol/L, multiply by 0.05551.

BMI: Body Mass Index

HOMA-IR, Homeostatic model assessment of insulin resistance

QUICKI: Quantitative insulin check index

G/I: Fasting glucose to fasting insulin ratio

DHEA-S: Dehydroepiandrosteronesulphate

FSH: Follicle stimulating hormone

LH: Luteinizing hormone

mmol/l: milimol per liter

µU/ml: micro Units per milliliter

mg/dL: microgram per deciliter

µg/l, microgram per liter

**Table II:** Clinical characteristics and serum hormone concentrations in women with the polycystic ovary syndrome after administration of insulin-sensitizing drugs for three months

**Variable **	**Metformin**			**Pioglitazone**			**Combination**			**p-value**
	**Pre** **treatment**	**Post** **treatment**	**p-value**	**Pre** **treatment**	**Post** **treatment**	**p-value**	**Pre** **treatment**	**Post** **treatment**	**p-value**	
Weight(kg)	71.3±11.2	71±12.8	NS	70.6±13.2	72.1±13.1	<0.001	72.6±9.4	71.5±9.8	NS	NS
BMI(kg/m^2^)	27.5±3.6	27.4±4.4	NS	27.2±4.7	27.8±4.7	<0.001	28.5±3.2	28±3.4	NS	NS
Waist-to-hip ratio	0.83±0.031	0.83±0.036	NS	0.80±0.055	0.81±0.055	0.001	0.82±0.045	0.82±0.049	NS	NS
Fasting glucose (mmol/l)	5.4±0.5	5.1±0.3	0.012	5±0.2	4.8±0.2	0.018	5.1±0.5	5±0.4	NS	0.02
Fasting insulin (µU/ml)	17.1±10.8	10.3±5.6	0.003	12.4±7.1	8.6±4.9	0.015	11.7±7.5	7.3±4.3	0.026	NS
HOMA-IR	4.2±2.7	2.3±1.2	0.001	2.8±1.6	1.8±1	0.011	2.7±1.8	1.6±0.8	0.013	NS
QUICKI	0.31±0.03	0.34±0.03	0.001	0.33±0.02	0.35±0.03	0.007	0.33±0.03	0.36±0.03	0.009	NS
Fasting G/I ratio (mg/dL per µIU/mL)	7.8±5.3	14.6±12.4	0.008	9.5±4.7	13.9±8.9	0.026	10.6±6.3	15.7±7.5	0.03	NS
LDL cholesterol (mg/dl)	131.8±26.7	127±28	NS	113.8±54.8	110±52.6	NS	126.9±33.5	124±29.8	NS	NS
HDL cholesterol (mg/dl)	51.1±12.1	52.9±11	NS	51.1±12.5	52.8±12.5	NS	52.2±12.2	55.7±13.2	NS	NS
Serum cholesterol (mg/dl)	215±36.5	209.7±37.2	NS	185.6±51.6	177.7±53.3	NS	196±36.6	197±35.6	NS	NS
Serum triglyceride (mg/dl)	140.3±58.2	147±78.9	NS	111.6±76	100.9±61.8	NS	131±59.3	114.3±78.5	NS	NS
DHEAS (mg/l)	1.5±0.7	1.4±0.5	NS	1.4±0.7	1.6±0.6	NS	1.4±0.7	1.5±0.8	NS	NS
Total testosterone (µg/l)	0.7±0.2	0.6±0.18	0.018	0.7±0.1	0.7±0.2	NS	0.6±0.2	0.6±0.1	NS	NS
FSH (IU/L)	5.2±1.7	5.5±1.4	NS	4.7±1.2	5.6±2.1	0.041	5.6±2.2	7±2.2	0.005	0.01
LH(IU/L)	7.9±2.3	6.3±2.4	0.002	7.6±2.9	6.3±2.4	NS	6.8±2.7	6.4±2.2	NS	NS
LH/FSH	1.6±0.6	1.1±0.4	0.002	1.6±0.8	1.1±0.4	0.07	1.4±0.7	1±0.5	0.003	NS

Note*: *Plus-minus values are means ± SD. To convert values for fasting glucose to mmol/L, multiply by 0.05551.

BMI: Body Mass Index

HOMA-IR: Homeostatic model assessment of insulin resistance.

QUICKI: Quantitative insulin check index

G/I: Fasting glucose to fasting insulin ratio

DHEA-S: Dehydroepiandrosterone sulphate

FSH: Follicle stimulating hormone.

LH: Luteinizing hormone

mmol/l: milimol per liter

µU/ml: micro Units per milliliter

mg/dL: microgram per deciliter

µg/l: microgram per liter

NS; Non-significant

LTFU=Lost to follow-up

WDFS=Withdrew from the study

**Figure 2:**
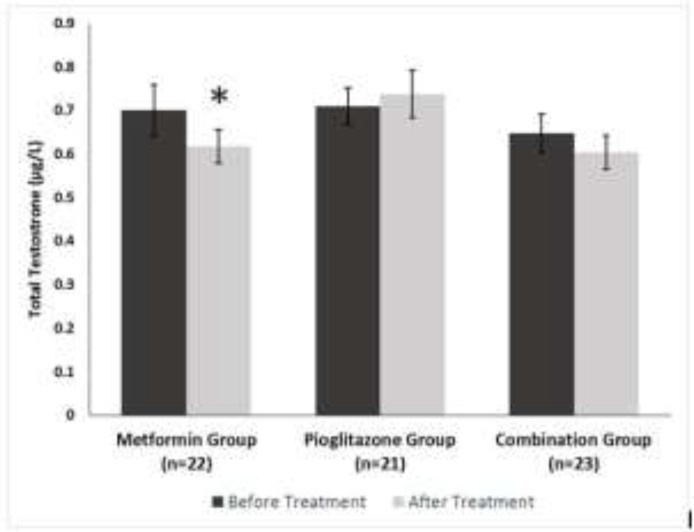
Serum total testosterone in women with PCOS at baseline and after three months of treatment. *= p<0.05(± SD

## Discussion

To our knowledge, the clinical, hormonal and metabolic change after treatment with metformin and pioglitazone has not been investigated in Middle Eastern women. In this study, we evaluated the efficacy of a combined therapy with metformin and pioglitazone as compared to metformin or pioglitazone monotherapy on the clinical and endocrine-metabolic alterations of PCOS patients. Specifically, we hypothesized that combined treatment would be more potent in treatment of symptoms and biochemical abnormalities characteristic of PCOS, compared to single-agent therapy. Metformin therapy has been shown to have beneficial effects on ovarian function, clinical feature, hormonal and metabolic parameter in women with PCOS (23, 36-37). It was shown that pioglitazone ameliorates the hormonal and metabolic consequence of PCOS (38-40). This trial purposed to contrast and compare the hormonal, metabolic and clinical alterations following treatment with metformin and pioglitazone alone and in combination in women with PCOS. 

This study illustrated insulin-sensitizing drugs exerted multiple beneficial effects on PCOS women with or without insulin resistance. Our investigation showed ameliorating in insulin resistance and hyperinsulinemia after administration of pioglitazone and metformin for a period of three months in PCOS women. We observed decrease of 44.5%, 33.3% and 41% in the HOMA-IR index after three months of treatment with metformin, pioglitazone and combination therapy, respectively, and decreases of 39.7%, 30.6% and 37.2% in fasting insulin levels after administration of metformin, pioglitazone and combination therapy, respectively. These variations of improvement in insulin resistance and insulin levels can be ascribed to the effects of metformin or pioglitazone. With regard to the role of insulin resistance as a component of metabolic syndrome and its relation to cardiovascular disease, this decrease can be useful in these patients.

We found that treatment with pioglitazone improved insulin action and insulin resistance despite an increase in body weight, waist to hip ratio and unfavorable BMI, which is in agreement with some previous studies using pioglitazone in PCOS women (41-43) or other TZDs in patients with DM2 and insulin resistance (44-45). Improvement in insulin sensitivity despite increase in body weight could be explained by the advantageous transference from visceral to subcutaneous fat and the concurrent amelioration insulin sensitivity of hepatic and peripheral tissue induced by TZDs (46-48). The reduction of lipid profile did not show significance difference probably because of the short duration of the study. Three women on metformin (10.7%), seven on pioglitazone (25%) and 3.5% on combination therapy became pregnant during the study. 

In this study, it appears that insulin sensitizing drugs might improve menstrual regularity compared to the baseline non-treated state, although this result was consistent with previous reports in women with PCOS taking either pioglitazone or metformin (21, 32, 38, 49). We found combination therapy as well as administration of pioglitazone for three months is more effective than metformin alone in ameliorating menstrual cyclicity. This finding is in line with Glueck *et al* who evaluated the effect of pioglitazone plus metformin diet on women who were non-optimally responsive to metformin diet alone (39). There was significant difference in body weight after treatment with pioglitazone and no significant change after combination therapy was seen; therefore it may be concluded that the improvement of menstrual cycles was not operated via the body weight loss.

Both in vitro and in vivo studies showed that hyperandrogenism in PCOS women might be a result of hyperinsulinemia from peripheral insulin resistance (48, 50, 51). In the present study, we observed that metformin compared to pioglitazone or combination therapy resulted in a significant decrease in the levels of total testosterone. Our findings were in contrast with Ortega-Gonzalez *et al* who assessed responses of serum androgen after treatment with metformin and pioglitazone in PCOS women (38). Also, our data were not in agreement with Legro *et al* who examined the effect of metformin and rosiglitazone, a member of the thiazolidinedione family like pioglitazone, on ovarian function (52). Another study showed that metformin was more effective in reducing testosterone levels but rosiglitazone had a better effect on decreasing serum insulin and insulin resistance (53).

The lack of change after treatment with pioglitazone in total testosterone levels in the present study could be elucidated in two ways: 1) more effect of metformin on the ovarian cells and preventing androgen production; 2) probably inadequate dose of pioglitazone was given. It is not clearly known whether the profitable effect of TZDs in women with PCOS is due to a direct effect on ovarian function or adrenal gland or is due to reduction in insulin resistance. However, according to the results of this study, it appears that the effect of these drugs in PCOS is due to improved insulin resistance.

In this study a higher pregnancy rate was occurred in the pioglitazone compared to the metformin group, nevertheless our sample size is small for any ultimate conclusions. It is necessary to do more studies on the safety of TZDs and metformin in women who may achieve pregnancy during treatment with these drugs. Metformin is classified as a pregnancy category B drug and seems to be safe during pregnancy. Nawaz *et al* reported that in PCOS women, the continued use of metformin during pregnancy decreases the rates of abortion, gestational diabetes and fetal growth limitation (54). Rosiglitazone and pioglitazone are classified as pregnancy category C drugs, so PCOS women treated with these drugs should be given contraceptive before treatment and cautiously instructed to stop using the drug instantly after confirmation of pregnancy (55, 56). However two reported cases of women who taking the rosiglitazone during pregnancy have shown no infants malformation (57, 58).

Unexpectedly, in contrast to our hypothesis, our data showed that combination therapy may have little advantages compared to treatment with each drug alone. Similar findings have been reported in women with PCOS treated with metformin and other TZDs. Baillargeon *et al* reported that combined administration of metformin and rosiglitazone do not illustrate noteworthy benefits over using alone in normal insulin sensitivity non-obese women with PCOS (27). They observed a considerable amelioration in ovulatory frequencies on metformin in comparison with rosiglitazone, but the combination was not more potent. After treatment unbound testosterone levels were comparable between three groups, and there was also no significant change in area under insulin curve and fasting insulin levels after administration of rosiglitazone compared to placebo, but there was a noticeable improvement after metformin or combination therapy. Huang *et al* showed metformin can be amend health-related quality of life of women with polycystic ovary syndrome by meliorate psychological disorders due to acne, hair loss and infertility problems, especially for hyperandrogenic patients who are overweight (59). Ghaneei *et al* reported, the outcomes demonstrated that cabergoline can be utilized as a protected organization for PCOS patients with hyperprolactinemia to enhance the menstrual cycles (60).

Legro* et al* reported no beneficial effects of combined rosiglitazone and metformin therapy over single-agent therapy. They showed more improvements with rosiglitazone compared to metformin on free testosterone levels, hyperinsulinemia, and trending toward elevated ovulation rate (52). Glueck *et al* reported that when pioglitazone was added to metformin therapy, in women with PCOS who failed to respond optimally to metformin, insulin resistance, fasting glucose, insulin level, and DHEAS significantly decreased and HDL cholesterol and sex hormone-binding globulin striking increased and menstrual cyclicity was improved (39). There are some pitfalls in our study. The primary flaws are the small sample size, short time of drug administration and restrictions to follow-up the subjects more than three months, so further large scale randomized trial with longer duration of drug use and longer follow-up period are required to assess effectiveness of the metformin and pioglitazone in women with PCOS.

In summary, we found that insulin-sensitizing drugs have favorable influence on menstrual cyclicity improvement and insulin resistance for women with PCOS. But metformin was more effective than pioglitazonein to improve hyperandrogenemia, serum insulin levels, and insulin-resistance; and pioglitazone was more efficient in regulating the menstrual cycle and clinical sign of hyperandrogenism (acne) and the adjunct of pioglitazone to metformin may not have further benefit compared to treatment with using drugs alone. This study might recommend that pioglitazone is a better choice in less-insulin-resistant women with PCOS who are seeking fertility or improvement in clinical signs of hyperandrogenism and modest increase in testosterone levels while in cases with predominant hyperandrogenemia and insulin resistance, metformin should be applied.

## Note

***Registration ID in IRCT: IRCT201403101836N7***
